# Higher order interaction analysis quantifies coordination in the epigenome revealing novel biological relationships in Kabuki syndrome

**DOI:** 10.1093/bib/bbae667

**Published:** 2024-12-19

**Authors:** Sara Cuvertino, Terence Garner, Evgenii Martirosian, Bridgious Walusimbi, Susan J Kimber, Siddharth Banka, Adam Stevens

**Affiliations:** Division of Evolution and Genomic Sciences, Faculty of Biology, Medicine, and Health, School of Biological Sciences, The University of Manchester, Manchester, UK; Division of Cell Matrix Biology and Regenerative Medicine, Faculty of Biology, Medicine, and Health, School of Biological Sciences, The University of Manchester, Manchester, UK; Division of Developmental Biology & Medicine, Faculty of Biology, Medicine, and Health, School of Biological Sciences, The University of Manchester, Manchester, UK; Division of Evolution and Genomic Sciences, Faculty of Biology, Medicine, and Health, School of Biological Sciences, The University of Manchester, Manchester, UK; Division of Developmental Biology & Medicine, Faculty of Biology, Medicine, and Health, School of Biological Sciences, The University of Manchester, Manchester, UK; Division of Developmental Biology & Medicine, Faculty of Biology, Medicine, and Health, School of Biological Sciences, The University of Manchester, Manchester, UK; Manchester Centre for Genomic Medicine, St. Mary’s Hospital, Manchester University Foundation NHS Trust Health Innovation Manchester, Manchester, UK; Division of Cell Matrix Biology and Regenerative Medicine, Faculty of Biology, Medicine, and Health, School of Biological Sciences, The University of Manchester, Manchester, UK; Division of Evolution and Genomic Sciences, Faculty of Biology, Medicine, and Health, School of Biological Sciences, The University of Manchester, Manchester, UK; Manchester Centre for Genomic Medicine, St. Mary’s Hospital, Manchester University Foundation NHS Trust Health Innovation Manchester, Manchester, UK; Division of Developmental Biology & Medicine, Faculty of Biology, Medicine, and Health, School of Biological Sciences, The University of Manchester, Manchester, UK

**Keywords:** Kabuki syndrome, KMT2D, DNA methylation, integration analysis

## Abstract

Complex direct and indirect relationships between multiple variables, termed higher order interactions (HOIs), are characteristics of all natural systems. Traditional differential and network analyses fail to account for the omic datasets richness and miss HOIs. We investigated peripheral blood DNA methylation data from Kabuki syndrome type 1 (KS1) and control individuals, identified 2,002 differentially methylated points (DMPs), and inferred 17 differentially methylated regions, which represent only 189 DMPs. We applied hypergraph models to measure HOIs on all the CpGs and revealed differences in the coordination of DMPs with lower entropy and higher coordination of the peripheral epigenome in KS1 implying reduced network complexity. Hypergraphs also capture epigenomic trans-relationships, and identify biologically relevant pathways that escape the standard analyses. These findings construct the basis of a suitable model for the analysis of organization in the epigenome in rare diseases, which can be applied to investigate mechanism in big data.

## Introduction

Omic datasets, such as epigenomics and transcriptomics, are usually interpreted using a differential analysis approach, which treats each variable independently. A complementary approach is a network-based analysis, which models biological systems as pairs of interacting variables [1]. Network-based models can identify core clusters of genes that are likely to be mechanistically relevant but can miss complex direct and indirect relationships [2] especially those between multiple variables [3].

Complex systems can rarely be captured by their pairwise dynamics alone [4,5]. Rather, natural systems demonstrate relationships between multiple variables [6] known as higher order interactions (HOIs). HOIs have been shown to mark biological phenomena such as stem cell development [7] and embryo implantation [8]. HOIs can be measured by hypergraphs, which are a generalization of a graph (network) in which an edge can join any number of vertices. Hypergraphs can reveal mechanistic insights that can escape traditional analyses [9–11], including the impact of both HOIs [4,12] and direct and indirect interactions. Importantly, hypergraph models are viewed as mechanistic and do not rely on qualitative assessment of gene ontology to establish function [9–11,13].

Elements of complex systems are coordinated by HOIs [10]. This coordination can be assessed using entropy [14], which measures network structure, combined with measuring network path directness. They have been used to examine early human embryology [7,15] and hypergraph topology has been used to assess mechanism in neural, ecological and social systems [12]. Recently, efficient imputation of multi-tissue and cell-type gene expression has been achieved using a hypergraph approach [16]. However, hypergraphs have not yet been used to assess mechanistic relationships in human diseases. In this study, we have tested the hypergraph approach in context of epigenomic data from a rare disease, Kabuki syndrome (KS) type 1.

**Figure 1:**
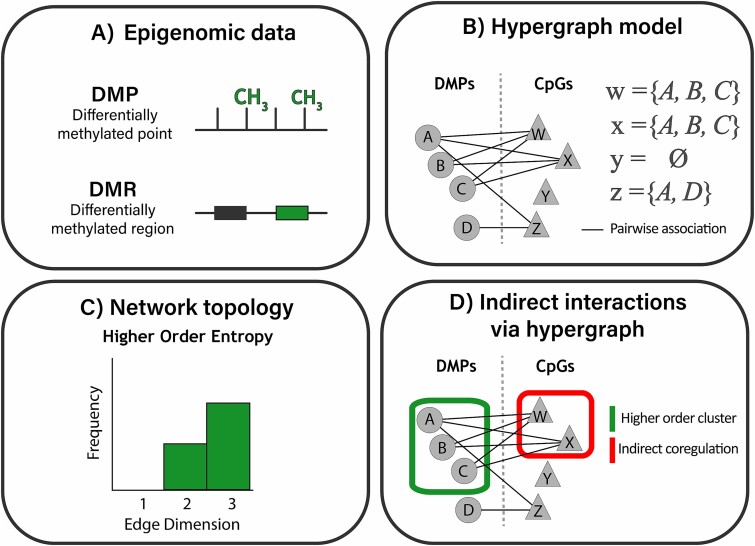
Experimental design to assess the coordination of peripheral blood DNA methylation associated with Kabuki syndrome. The core features of the experimental design used to assess differences in the coordination of the epigenome between KS1 and controls. A) Peripheral blood DNA methylation is used to identify differences in the epigenome between KS1 and controls. Differentially methylated points (DMPs) and regions (DMRs) are defined using a statistical approach. B) Pairwise associations are defined between DMPs (A-D) and all remaining CpGs (W-Z). These bipartite network models distinguish HOIs from pairwise associations in the epigenome. Associations with CpGs which are common between DMPs can be considered HOIs between DMPs, or hyperedges, described here by sets w-z. C) To measure coordination of these higher order networks, an indicator of function, Shannon entropy is calculated on the distribution of edge dimensions. D) Hypergraph models are generated from these bipartite structures and refine clusters of coordinated DMPs (green box) as well as implicating a wider set of CpGs as potentially indirectly co-regulated (red box).

KS is one of the commonest Mendelian histone lysine methylation disorders [17]. Most KS cases are caused by heterozygous loss-of-function variants in H3K4 methyltransferase 2 D (*KMT2D)* (Kabuki syndrome type 1 [KS1], OMIM#147920), while less than 5% of cases are caused by X-linked *KDM6A* variants (KS2, OMIM#300867) [18–20]. By regulating enhancer and promoter elements, KMT2D controls the access of transcription factors and other proteins to DNA and therefore controls gene transcription and regulation [21–25]. Clinically, KS is characterized by distinct facial dysmorphism, intellectual disability, developmental delay, and a range of internal organ malformations such as congenital heart defects, skeletal defects, cleft palate and genitourinary malformations [26]. In addition, affected individuals are susceptible to several functional anomalies (e.g., endocrine disorders, deafness) and immune defects [26–28].

DNA methylation can act in both a cis- and trans-regulatory manner in the control of gene expression [29], however even those studies which investigate trans-interactions do not consider higher order effects. Here, we have integrated KS1 and control genome-wide DNA methylation data from three studies to identify novel differentially methylated CpGs (differentially methylated points [DMPs]) and differentially methylated regions (DMRs). To identify HOIs in the KS1 epigenome we have generated hypergraph models of observed methylation patterns, assessed epigenomic coordination by quantifying the entropy of those network models, and investigated the directness of the relationships between epigenomic points defined by them (Fig. 1). We reveal multiple novel, biologically relevant relationships in KS1 utilizing a methodology which captures the HOIs of complex systems more completely [30] and is therefore better able to capture causally relevant associations [31–33]. Overall, we show that hypergraphs allow investigation of the entire epigenome, including coordination over genomic distance, in the context of a rare disease, and provides a new quantifiable framework to investigate causal relationships.

## Material and methods

### Data acquisition

Peripheral blood DNA methylation data from individuals with KS1 and controls was obtained from previously published studies namely Butcher *et al.*, Sobreira *et al.* and Cuvertino *et al.* [34–36]. Of note, we only included data from individuals with variants that could be definitely predicted to cause KS1. Specifically, from the dataset of Butcher *et al*. [35], we excluded 32 individuals with CHARGE syndrome (OMIM#214800) [11]. From the dataset of Sobreira *et al.* [36], we excluded two individuals with *KMT2D* variants of uncertain significance and six individuals with other variants. From the dataset of Cuvertino *et al.* [34], we excluded four individuals with exon 38 and 39 missense *KMT2D* variants that cause a syndrome that is genetically, epigenetically, and phenotypically distinct from four KS1 individuals.

### Data processing

All statistical analyses were performed in R 3.4.1 (www.r-project.org) [37]. The studies reported by Butcher *et al*. (GSE97362) and Sobreira *et al.* (GSE116300) were performed using Illumina Human Methylation 450 K (hereafter called 450 K arrays) [35,36] and Cuvertino *et al.* used Infinium Methylation EPIC bead chip (Illumina) [34]. In order to make the datasets comparable we removed all non-matching probes between the EPIC and 450 K arrays using the Bioconductor package *minfi* [38]. Distribution of cell types within the whole blood samples were estimated and adjusted using the established Houseman method for each individual [39]. Initial data quality was measured by removing the methylated and unmethylated background signal levels exceeding the detection threshold of P > 0.01. In addition, to minimize the differences between the samples, we excluded cross-reactive probes [40], probes on sex chromosomes and those that are age-associated [41–43]. Raw beta values were logit transformed to M values following functional and quantile, within array, normalizations (SWAN) [44]. We used a z score to normalize data for the study.

### Identification of significant DMPs and DMRs in KS1

Methylation data were analysed using the ChAMP [45] pipeline in R 3.4.1. DMPs were identified using a linear modelling approach to identify differentially methylation levels between groups of samples. DMRs were identified using the Bumphunter [46] method which defined DMRs as genomic regions with more than seven DMPs, with a maximum gap of 300 bp between DMPs. This technique performs a randomized permutation approach to identify regions with greater than expected number of DMPs. A false discovery rate modified *P* < 1 × 10^−4^ was used to identify significantly DMPs between KS1 individuals and controls. Z-scores of all methylated positions (3,166 probes) were analysed using Qlucore Omics Explorer 3.4 (Qlucore, Lund, Sweden). Principal components analysis (PCA) was used to visualize clustering of the samples based on DMPs between groups. A heatmap was generated using R package heatmap.2 to examine clustering of DMPs using the Euclidean metric. Relation to the island distribution graph was generated in R 3.4.1.

### Statistical analysis

Significant differential methylation was defined as those CpGs with a false-discovery rate modified (FDR) *P* < 1 × 10^−4^. FDR correction was done using the Benjamini-Hochberg method on probes which passed the significance threshold individually. When assessing contribution of those DMPs to DMRs, P-values represent the percent of permuted regions with more extreme methylation than the null distribution; the family-wise error rate (FWER) values represent the proportion of permutations with at least one region with more extreme methylation than the null distribution [38,46].

When interpreting analyses of direct paths present in the network structures, Wilcoxon and Fisher’s exact tests were used. Wilcoxon tests were performed to assess the significance of differences between distributions; Fisher’s exact tests were used to assess differences in contingency tables.

We used a Bayesian approach to model the differences in entropy between hypergraph structures in pathways identified as being significantly enriched between KS1 and controls. We iterated 100 hypergraphs each, from genes attributed to the significant pathways and calculated entropy on each of these networks. The distribution of entropy values was resampled 10,000 times and the difference between posterior distributions was compared. When the 89% credible interval of the difference between the two distributions did not include 0, the difference was defined as significant. Results were plotted for pathways demonstrated to be significant.

### Gene ontology and epigenetic landscape

CpGs in 450 K and EPIC arrays were annotated using the method incorporated into the ChAMP pipeline, utilizing Infinium HumanMethylation450 v1.2 and Infinium MethylationEPIC v1.0 datasets from Illumina, respectively, each curated with UCSC Genome Browser annotations. Gene ontologies associated with DMPs, DMRs and elements refined from the hypergraph approaches were assessed using WEB-based Gene SeT AnaLysis Toolkit (WebGestalt 2019) [47]. UCSC Genome browser database was used to study the epigenetic landscape (H3K4me1, H3K4me3 and H3K27ac) of selected DMRs [48] in human blood cells (B cell, CD133HSC, Neutrophils) (Bernstein Lab, Broad Institute, ENCODE consortium).

### Hypergraph analysis

A hypergraph is a generalization of a network structure wherein an edge can connect an arbitrarily large number of vertices. This enables hypergraphs to capture associations between multiple factors, believed to be important in defining causality in complex, dynamic and possibly non-deterministic systems [31–33]. Hypergraph models were generated here to model the relationships between a target set of epigenetic variables (as vertices), considering the backdrop of broader epigenetic variation (as edges) [9–11,30]. These analyses were performed separately for controls and KS1 to produce separate hypergraph models of healthy and disease methylome network structure. Due to the highly multivariate nature of the hypergraph models, we have not limited our approach to assessing cis-regulatory associations only.

Correlations were calculated between DMPs that distinguish KS1 from controls, and all other CpGs, which are not differentially methylated, using R (v3.4.1 [37]). To generate the hypergraphs, these correlation matrices were binarized using an R cut-off equal to the standard deviation of the absolute correlation values, such that only larger (positive or negative) correlations are retained.

The resulting matrix $(M)$ is a bipartite network (Fig. 1B) which describes pairwise associations between DMPs and all remaining CpGs. This also represents the incidence matrix of the hypergraph, where correlations shared between DMPs represent hyperedges connecting DMPs as vertices in the hypergraph, identifying groups of DMPs whose methylation patterns are coordinated with thousands of other CpGs. This matrix was then multiplied by the transpose of itself $\left({M}^t\right)$ to give the final matrix $\left(M\cdotp{M}^t\right)$ whose values describe the number of correlations any pair of DMPs share across the methylome; this represents the adjacency matrix of the hypergraph [49,50].

By hierarchical clustering of the hypergraph adjacency matrix, represented using a heatmap, a primary cluster of DMPs can be refined, connected by a large number of hyperedges. The specific hyperedges connecting groups of DMPs are CpGs which can be identified from the incidence matrix, to associate a wider element of the methylome with KS1. To do this, we refined the incidence matrix of the hypergraph to a set CpGs which are associated with >95% of the target DMPs.

### Non-negative matrix factorization

Non-negative matrix factorization (NMF) clusters the correlation matrix of DMP coordination based on the contribution of each DMP to an underlying linear model [51]. This approach can be used to measure coordination between DMPs and represents a computationally intensive validation of the hypergraph approach presented in this study.

A correlation matrix was generated between the target DMPs and the remaining CpGs, limited to the 100,000 CpGs with the smallest P-values due to computational constraints. NMF was performed and the resulting matrix was clustered to refine sets of DMPs with similar relationships to one another when compared to the remaining CpGs. We investigated the overlap between clusters generated by NMF and those generated by hypergraph approaches.

### Entropy

Entropy is a mathematical property quantifying organization. In information theory, Shannon entropy can be used to describe the range of expected outcomes, expressed as a probability distribution of the outcomes; in network analysis, this can be equated to the distribution of connections between vertices. In gene regulatory networks, connections between vertices represent the existence of possible associations between genes; hypergraphs allow us to more completely capture those associations compared to pairwise network approaches and entropy provides a metric by which we can describe the structure of the resulting network. Network entropy has been demonstrated as informative in defining cellular differentiation potential [52] and in inferring causal relationships between network elements [53]. Specifically, high entropy describes more uniform networks and has been associated with less differentiated cellular states [52].

Shannon entropy was calculated within the central cluster of the adjacency matrix of each hypergraph. The values were compared to those calculated from hypergraphs of randomly selected CpGs, to assess whether coordination of DMPs was different to the rest of the methylome. This was iterated 1,000 times for both KS1 and controls.

### Network silencing

To assess the underlying map of DMP coordination, analyses were performed to refine the direct elements from the hypergraph structure. This approach uses matrix transformation to discriminate direct from indirect associations and potentially refines functional or causal relationships [2] between network elements. We utilized the method introduced by Barzel and Barabási (Supplementary Formula 1), to silence indirect connections in the hypergraph structure. We then converted the remaining values into Z-scores to assess the distribution of direct network connections in KS1 and controls.

Having assessed differences between the directly connected elements in KS1 and controls, we identified elements which were present uniquely in each group. These unique elements were assessed for associations with known biology via gene ontology analyses. Finally, we assessed the proximity of DMPs defined as directly associated to determine the distribution of cis- versus trans-regulatory associations. We defined cis-associations between DMPs as those where the elements were less than 1 Mb apart; elements more distant than this were labelled trans-associations.

## Results

### DMR analysis using integrated dataset identifies new biological processes disrupted in KS1

**Figure 2:**
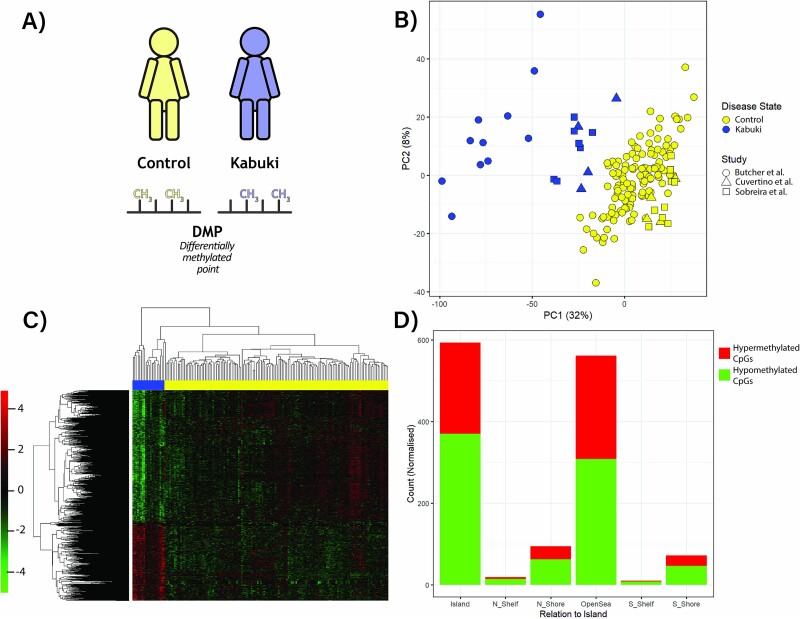
Peripheral blood DNA methylation reveals differences between KS1 and controls. A) Differentially methylated points are identified between KS1 and control patients. Control samples are indicated in yellow, KS1 in blue. B) Principal component analysis (PCA) of the peripheral blood DNA methylome demonstrates variation between KS1 and control samples. Shapes are used to differentiate the studies from which data were drawn: circles represent samples from Butcher et al., squares represent Sobreira et al. and triangles Cuvertino et al. C) a heatmap of relative methylation of DMPs (FDR).

We set out to identify the DMRs in KS1 compared to controls. We identified 417,217 common probes across the three peripheral blood DNA methylation array datasets to enable comparison between data from 22 individuals with KS1 and 138 controls (Fig. 2A). PCA analysis performed using only DMPs demonstrated segregation of KS1 individuals from controls suggesting robustness of our data integration (Fig. 2B).

From this dataset, we identified 2,002 DMPs in the KS1 samples compared to the controls (FDR < 1 × 10^−4^). Of these, in KS1 753 DMPs were hypermethylated and 1,249 were hypomethylated (Fig. 2C). Our analysis identified more DMPs compared to previous studies [35,36]. DMPs were found to be located predominantly in CpG islands and open sea (4 Kb from CpG islands) regions (Fig. 2D).

**Figure 3:**
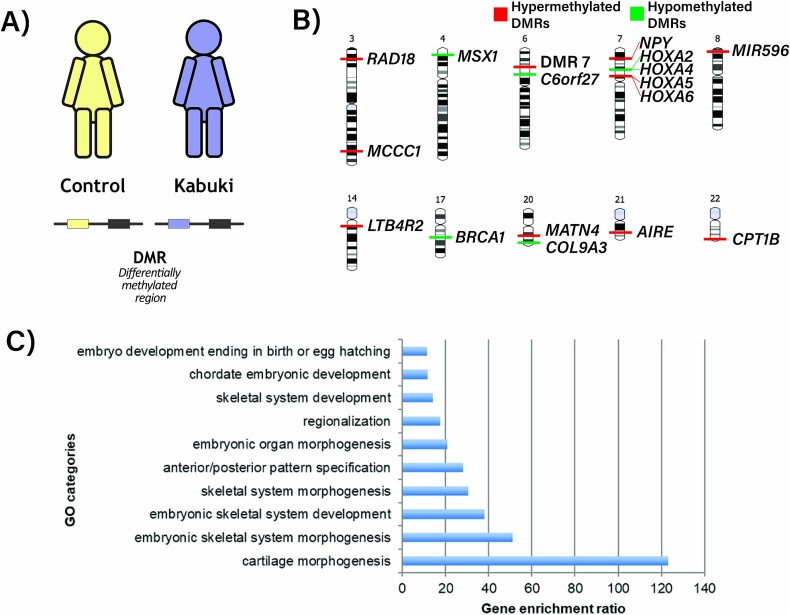
KS1 results in differences in methylated regions between KS1 and controls. A) Differentially methylated regions are identified between KS1 and control patients. Control samples are indicated in yellow, KS1 in blue. B) Ideograms show the 17 DMRs (FWER<0.05).

Using these DMPs, we identified 208 DMRs (> 7 annotated DMPs) between KS1 and controls (87 hypermethylated and 121 hypomethylated, FDR < 1 × 10^−3^) (Fig. 3A). Of these, 17 DMRs (11 hypermethylated and six hypomethylated) passed the cut-off of FWER<0.05 (Table 1) (Fig. 3B). Our analysis validated three previously identified DMRs (*HOXA4, HOXA5, HOXA6*) [35,36] for KS1, and identified 14 additional DMRs, even though the cut-offs used here were more stringent than the previous studies [35,36].

**Table 1:** List of DMRs including chromosome (Chr), gene annotation, relative methylation value, P-value and family wise error rate (FWER) corrected P-value. DMRs with FWER<0.05 are included.

**DMR**	**Chr**	**Genes**	**Value**	**P-value**	**FWER**
DMR_1	chr7	*HOXA5*	7.87	<2.2 × 10^-16^	<2.2x10-16
DMR_2	chr14	*LTB4R2*	8.76	<2.2 × 10-16	<2.2 × 10-16
DMR_3	chr8	*MIR596*	9.88	<2.2 × 10-16	<2.2 × 10-16
DMR_4	chr7	*HOXA4*	-6.65	<2.2 × 10-16	<2.2 × 10-16
DMR_5	chr7	*HOXA6*	8.16	<2.2 × 10-16	<2.2 × 10-16
DMR_6	chr7	*HOXA2*	-6.26	<2.2 × 10-16	<2.2 × 10-16
DMR_7	chr6	*–*	6.36	6.86E-06	0.001
DMR_8	chr7	*NPY*	7.55	4.80E-05	0.001
DMR_9	chr4	*MSX1*	-6.64	5.72E-05	0.001
DMR_10	chr20	*MATN4*	6.33	8.23E-05	0.002
DMR_11	chr17	*BRCA1*	-6.01	8.23E-05	0.005
DMR_12	chr22	*CPT1B*	5.98	1.23E-04	0.008
DMR_13	chr6	*C6orf27*	-5.86	8.46E-05	0.012
DMR_14	chr21	*AIRE*	6.18	1.67E-04	0.02
DMR_15	chr3	*RAD18*	84.13	9.60E-05	0.041
DMR_16	chr20	*COL9A3*	-4.47	1.03E-04	0.045
DMR_17	chr3	*MCCC1*	9.30	1.23E-04	0.05

We then performed gene ontology analysis using the 17 significant DMRs. This showed genes within these regions to be associated with embryonic skeletal system morphogenesis and development (*HOXA2, HOXA4, HOXA5, HOXA6, MSX1, MATN4, COL9A3*), anterior/posterior pattern specification and embryonic organ morphogenesis (*HOXA5, HOXA4, HOXA6, HOXA2, MSX1*) (Fig. 3C).

Next, using Encode data we analysed the epigenetic landscape of the DMRs in human blood cells (B cells, CD133 hematopoietic stem cells, and neutrophils) using the UCSC genome browser (Supplementary Fig. S1 and Supplementary table 1). These cells were chosen because our DNA methylation dataset is from peripheral blood DNA samples. Seven out of 17 DMRs showed H3K4me3 and H3K27Ac marks indicating transcriptional activity in these cells. As DNA methylation regulates gene expression, we also evaluated the level of expression of the genes corresponding to the identified DMRs in RNAseq datasets (GSE126167, GSE126166) [54] from human or mouse KS1 cell models. In murine HT22 cells with homozygous deletion of the catalytic SET domain of *Kmt2d*, *Hoxa2,* and *Aire* were differentially expressed while *Npy* and *Col9a3* were down-regulated in EdU+ DG nuclei of *Kmt2d*^+/−βgeo^ mice compared to controls [54].

Collectively, these results reveal evidence of disruption of several disease relevant biological processes in peripheral blood derived epigenome of KS1, and thus demonstrate the power of data integration in revealing novel mechanistic insights.

### Hypergraph analysis identifies differences between biological pathways enriched in KS1 and control DMPs

Although informative, the 17 significant DMRs collectively represent only 189/2,002 significant DMPs. Hence, the DMR analysis ignored the functional relevance of >90% (1,817 out of 2,002) of the significant DMPs. This is especially true for DMPs present in open sea regions, where CpGs and probe density are lower, thus reducing the chances of identifying DMRs (Fig. 2D). We, therefore, wanted to interrogate the mechanistic relevance of all significant DMPs. However, methylation status of DMPs or CpGs could directly or indirectly affect the methylation status of other DMPs or CpGs. In other words, DMPs are likely to have a complex coordination with other DMPs and other CpGs. Hence, treating DMPs as independent variables and simply looking for enrichment of genes in the DMP datasets could be misleading. We, therefore, employed a hypergraph approach to identify groups of DMPs exhibiting HOI utilizing the entire epigenomic dataset [10,11,13]. In this context, the hypergraph summarizes correlations between each DMP and all remaining CpGs to infer relationships between DMPs; larger numbers of shared correlations between DMPs indicate higher order relationships.

**Figure 4:**
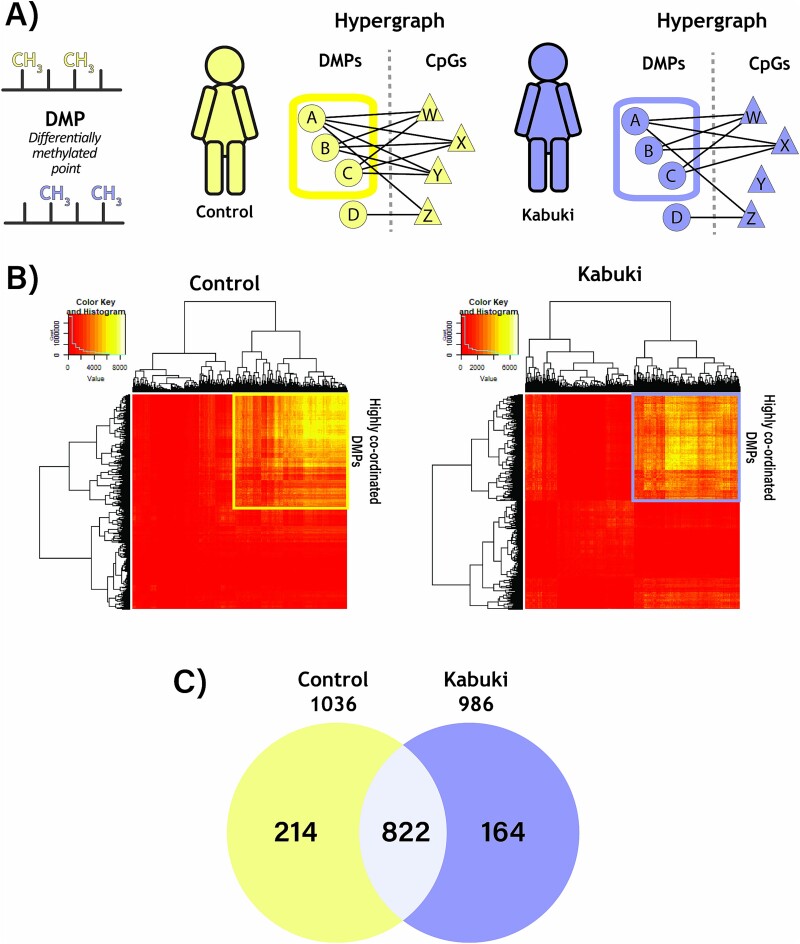
Clustering hypergraph adjacency matrices reveals HOIs between DMPs which distinguish KS1 and controls. A) Pairwise relationships between DMPs and CpGs can be summarized as HOIs between pairs of DMPs using a hypergraph approach. B) Heatmaps of hypergraph adjacency matrices for control (L) and KS1 (R). Red to yellow coloring in the heatmap represents increasing dimensionality of the hyperedges between pairs of DMPs. Hierarchical clustering of the hyperedges reveals a central cluster of highly coordinated DMPs in controls (yellow box) and KS1 (purple box) associated to one another by HOIs. C) Venn diagram demonstrating the overlap of DMPs in the central cluster of the hypergraphs.

Applying this hypergraph approach showed that 986/2,002 DMPs in KS1 and 1036/2,002 DMPs in controls were highly coordinated (appearing as heatmap clusters in Fig. 4A and4B). This means that methylation of these DMPs in KS1, or in controls, is highly interrelated. These results were validated using an independent approach of non-negative matrix factorization (Supplementary Fig. S2). Interestingly, 822/2,002 highly coordinated DMPs were found to be shared across both KS1 and control samples. This implies that 164/986 KS1-exclusive DMPs represent likely new disorder-specific interactions and 214/1,036 controls DMPs represent the interactions that are likely lost in KS1 (Fig. 4C).

Next, we performed gene ontology analysis on the 986 KS1 coordinated DMPs and identified an enrichment for genes (*P* < .05, FDR > 0.05) associated with connective tissue development (*TGFB1*), cartilage development (*TGFB1*) and neuronal migration (*TGFB1, NAV1*). In contrast, gene ontology analysis performed on the 1036 control coordinated DMPs demonstrated an association with response to interleukin-17 and vitamin D (Supplementary Fig. S3).

To further explore the functional relevance of DMPs and CpGs identified in hypergraph models, we investigated expressional changes of genes representative of these locations in previously published RNAseq datasets (GSE126167, GSE126166) of KS1 (Supplementary Fig. S4). Complex patterns of expression were observed between samples, with wild-type samples showing gene expression levels distinct to those in KS1, both in genes which were DMPs in our study as well as those which were not differentially methylated (wider associated CpGs). This finding supports the hypothesis that relevant associations may exist outside of differentially methylated regions of the chromosome and suggests that complex patterns of methylation may be reflected in gene expressional patterns.

Collectively, these results demonstrate the utility of hypergraph analysis to identify biologically relevant pathways from DNA methylation data that can escape the standard DMR-based analysis.

### Shannon entropy analysis shows differences in the network topologies of KS1 and control DMPs

**Figure 5:**
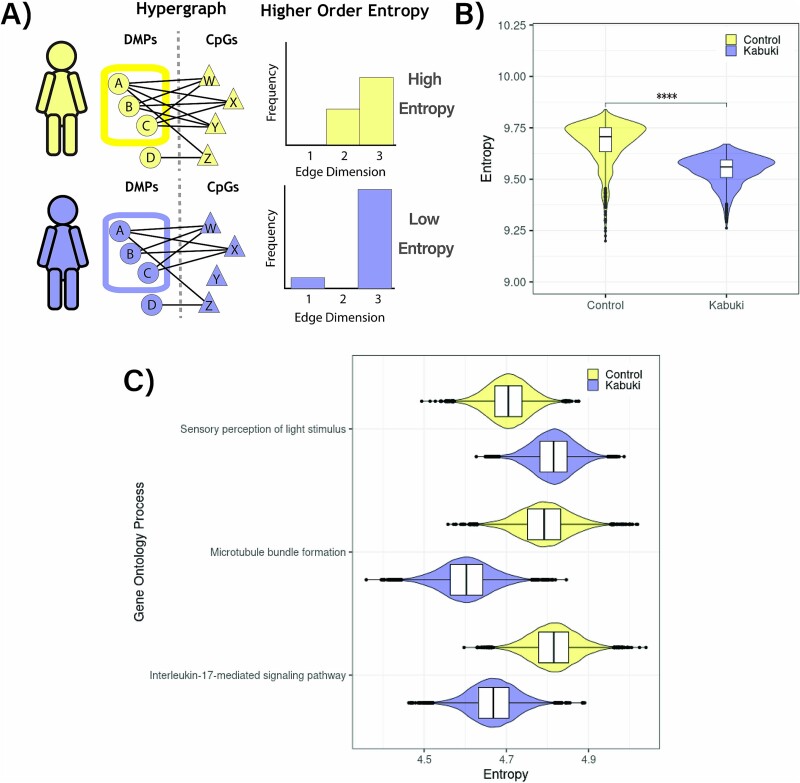
Topology of control and KS1 hypergraphs highlight differences in coordination. A) Co-ordination of the central cluster of the hypergraph in control (yellow) and KS1 (purple) can be quantified using Shannon entropy. Edge dimension is the number of shared correlations between a pair of vertices in the hypergraph model; entropy quantifies network structure from the distribution of edge dimensionality, such that a highly uniform network would have low entropy. B) Shannon entropy of the hypergraph central cluster of control and KS1 methylome. Presented data are compared to data from 1,000 matched iterations. C) Difference in entropy for pathways identified as enriched in the central clusters of either the control or KS1 hypergraphs. Bayesian Markov chain Monte Carlo sampling was performed to enable comparison between control and KS1. Only significantly different pathways (those where the 89% credible interval of the difference does not contain 0) are presented.

The hypergraph analysis showed that a large proportion of the clusters highly coordinated DMPs (822 DMPs) were shared between KS1 and controls, implying that although these CpGs are significantly differentially methylated in KS1, they remained highly coordinated in both KS1 and controls. Although the DMPs may remain highly coordinated in both sets, the nature (i.e., the direction or the strength of interactions) of these coordinations may be different in KS1 and controls. These differences are represented in the hypergraph model as the ‘network topology’. Differences in network topology can be measured using Shannon entropy [52], which examines the distribution of the number of shared correlations between any pair of DMPs (hyperedge dimensionality) in the hypergraph model (Fig. 5A). We, therefore, performed an iterative analysis of comparing Shannon entropy within the central cluster of the adjacency matrix and randomly selected CpGs of hypergraphs with 1,000 randomly generated simulations of 100 DMPs. We observed a lower entropy in the KS1 epigenome compared to controls, implying more ordered DMP coordination in the disorder (Fig. 5B).

Next, we assessed the contribution of the pathways enriched in the hypergraph central clusters (Supplementary Fig. S3) to the overall epigenome entropy using a Bayesian approach (Supplementary Fig. S5). This analysis shows that three pathways which were differentially enriched in the hypergraph central clusters have significantly different network entropy in KS1 and controls – ‘sensory perception of light stimulus’ (higher entropy in KS1) and ‘microtubule bundle formation’ and ‘Interleukin-17-mediated signaling’ (lower entropy in KS1) (Fig. 5C). Importantly, none of these pathways were identified using the traditional approach of one-to-one comparison of DMPs or DMRs.

This analysis shows that the KS1 coordinated epigenome has a different network topology compared to controls suggesting a more ordered and less diverse coordination by the DMPs in KS1.

### Indirect association of coordinated DMPs is distinct in KS1 compared to controls in the hypergraph models

**Figure 6:**
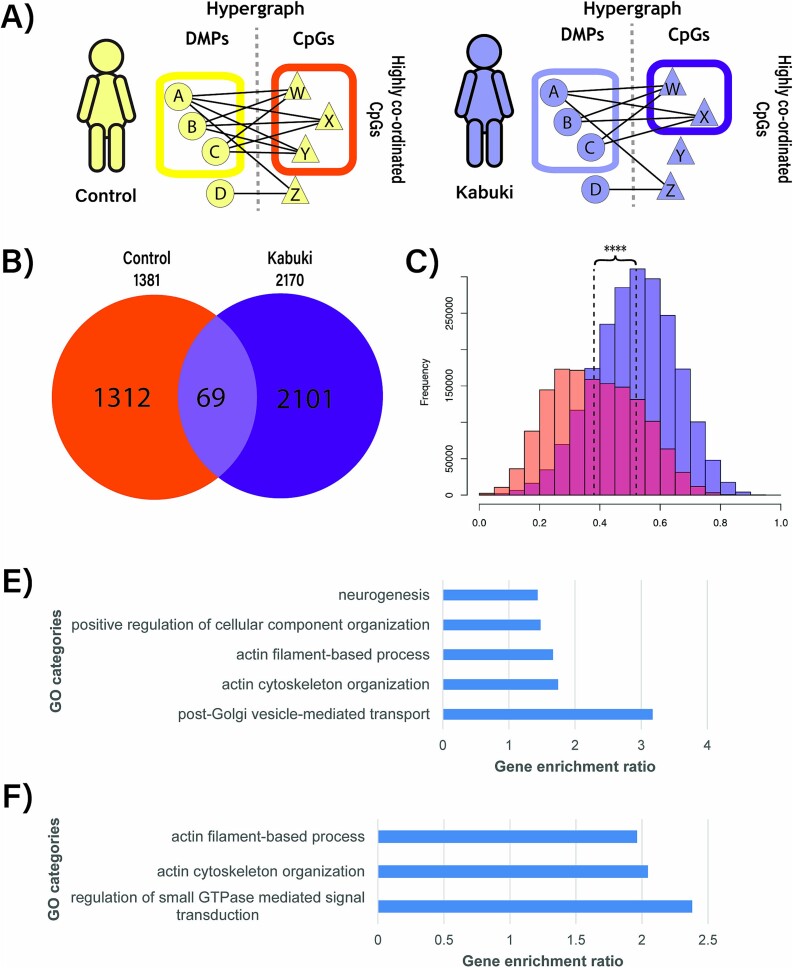
Co-ordinated DMPs demonstrate potential indirect co-regulation of a wider set of CpGs. A) In addition to refining clusters of coordinated DMPs (yellow/purple box) the hypergraph approach also implicates a wider set of CpGs (orange/dark purple box) as potentially indirectly co-regulated with the coordinated DMPs (yellow/purple box). B) Venn diagram of the indirectly co-regulated CpGs to demonstrate overlap between control and KS1. C) Distribution of correlation values (|r|) between DMPs and indirectly regulated CpGs in control (orange) and KS1 (dark purple). D-E) Ontology of nearest gene to indirectly regulated CpGs which were unique to control (D) or KS1 (E) (WebGestalt, FDR).

The results presented in this manuscript so far are based on analysis of DMPs. However, there may be disorder relevant to differences in the coordination of regions of the epigenome even if individual points may not be significantly differentially methylated. The hyperedges [10] in these models represent all coordinated CpGs within the entire epigenome, including those that are peripheral and not just the DMPs (i.e., the set of CpGs being coordinated by higher order action), thus capturing indirect associations in the epigenome (Fig. 6A). We, therefore, quantified the peripheral associations for KS1 and for controls. We detected more peripheral associations in KS1 compared to controls (2,170 and 1,381 CpGs respectively) with minimal overlap between the identified genes (Fig. 6B).

To quantify the peripheral associations present within KS1, we observed the strength of correlation values ($\left|r\right|$) between DMPs and CpGs which had been used to define hyperedges in the hypergraph models. More direct relationships between methylation of a DMP and a CpG would be reflected in a stronger correlation $\left|r\right|.$Comparing these data, we observed that the correlation values in KS1 were 1.3-fold higher compared to controls (*P* < 1 × 10^−9^) (Fig. 6C). Peripherally related CpGs in KS1 were enriched for gene ontologies related to small GTPase signal transduction (FDR < 0.01) (Fig. 6E) indicating that these indirect associations are likely gained in KS1. Peripherally related CpGs in controls were enriched for vesicle mediated transport and neurogenesis (FDR < 0.01) (Fig. 6D) indicating that these indirect associations are likely lost in KS1. Gene ontologies related to actin cytoskeleton organization were enriched in both KS1 and controls.

These results highlighted a greater variety of indirect associations of CpGs in KS1 compared to controls, implying major functional shifts in the wider epigenome between KS1 and controls.

## Discussion

**Figure 7:**
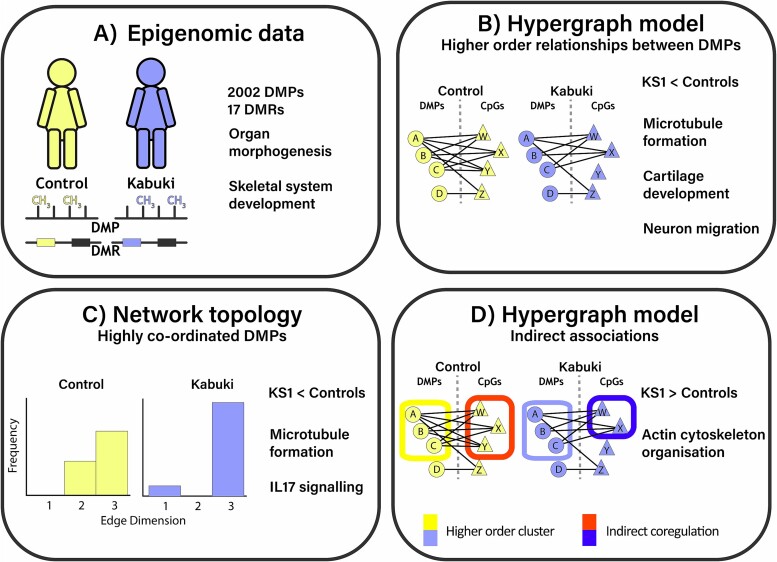
Analysis of blood DNA methylation reveals single CpG and regional differences, as well as differences in the coordination of the epigenome between KS1 and control. A) Statistical analysis highlights differences between KS1 and control in methylation of genes associated with morphology. B) HOIs between DMPs reveal a cluster of coordinated DMPs, present in both KS1 and control, in genes associated with organ development. C) Quantification of the coordination of DMPs demonstrated a lower entropy and therefore more defined coordination between DMPs in KS1 than control. D) The coordinated DMPs were indirectly associated with a wider range of CpGs in KS1 than controls. Differences in coordinated DMPs and indirectly implicated CpGs were associated with development and cellular organization.

We have analysed the impact of HOIs within the epigenome in a rare disease context to aid the understanding of disease mechanism (summarized in Fig. 7). Using the DMR based analysis, DMPs-based hypergraph, entropy studies, and entire epigenomic based indirect coordination analysis we identified several disease relevant genes, regions and pathways with mechanistic links.

Four DMRs correspond to *HOXA* genes, which play a fundamental role in embryonic development and in the anterior to posterior pattern specification [55] (Fig. 3). Two HOXA DMRs, corresponding to *HOXA4* and *HOXA2,* were found to be hypomethylated and two corresponding to *HOXA5* and *HOXA6* were hypermethylated*.* HOXA2 is involved in the dorsal-ventral patterns of neuronal development in the rostral hindbrain [56] and *HOXA2* variants cause microtia, hearing impairment and cleft palate (OMIM 612290) [57,58]. *HOXA4 is* involved in the anterior transformations of the dorsal aspects of components of the vertebral column [59]. HOXA5 is important in the patterning of the cervico-thoracic region and regulates organ development such as that of fore limb specification, cartilage, lung and gut [60]. HOXA6 is also important for hematopoietic cell proliferation and self-renewal [61]. We detected hypermethylation in the DMR corresponding to *MSX1* (Fig. 3), which has roles in limb-pattern formation and craniofacial development, particularly odontogenesis [62]. *MSX1* variants cause Witkop type ectodermal dysplasia 3 (OMIM 189500), orofacial cleft type 5 (OMIM 608874) and selective tooth agenesis (OMIM 106600) [63,64]. Dysregulation of these genes might explain the broad range of organ malformations and anomalies such as congenital cardiac defects, renal malformations and dental anomalies observed in KS1 [26].

We observed hypomethylation of DMR representing *NPY* (Fig. 3), which encodes a neuropeptide that is widely expressed in the central nervous system and influences many physiological processes, including cortical excitability, food intake, and cardiovascular function [65,66]. Gene ontology analysis of coordinated DMPs in KS1 identified DMPs representing *TGFB1, NAV1* (Fig. 3) that are involved in neuronal migration. We detected loss of coordination in peripherally related CpGs to be enriched for vesicle mediated transport and neurogenesis in Kabuki. Thus, dysregulation of these genes and pathways and processes may contribute to the neurodevelopmental and neurological phenotype of KS1 such as intellectual disability, with visuospatial construction, perception, memory, and language impairment [67].

Hypermethylation of *LTB4R2* (Fig. 3), a low-affinity receptor for leukotrienes [68], and hypermethylation of *AIRE,* a transcriptional regulator that interacts with the transcriptional coactivator CREB protein [69] were observed. These genes will be good targets to interrogate the mechanism of immune phenotypes in patients with KS1. We also detected loss of coordination and lower entropy of DMPs representing interleukin-17 in KS1. Dysregulation in these genes and pathways may contribute to the immune phenotypes of KS1 such as increase infection susceptibility and hypo-gammaglobulinemia [70].

We identified DMRs corresponding to *COL9A3* and *MATN4* (Fig. 3), which were hypo and hypermethylated, respectively. COL9A3 is a structural component of hyaline cartilage and the vitreous of the eye [71]. MATN4 is part of the extracellular matrix of various tissues in particular cartilage [72]. *COL9A3* variants cause Stickler syndrome type VI (OMIM 620022) characterized by hearing loss and skeletal abnormalities [73,74]. Dysregulation in these genes and pathways may contribute to the skeletal defects and joint hypermobility that are frequent in KS1.

The mechanism of generation of the abnormal DNA methylation pattern in individuals with KS1 remains to be examined. It could be a direct consequence of altered histone modifications and increased or decreased access to DNA methyltransferases and demethylase enzymes. Of note, KMT2D marks H3K4 in active enhancer regions, whereas CpG islands usually correspond to gene promoter regions. Hence, this raises alternative possibilities, such as that DNA methylation changes may be a secondary consequence of alterations in wider chromatin architecture or the 3D genomic architecture. It is also possible that these changes may be a consequence of subtle changes in cellular signaling, leading to alterations in developmental trajectories. It is notable that analysis based on DMRs derived from blood cells revealed dysregulation of genes and pathways that may contribute to non-blood phenotypes. Interestingly, we detected the greatest changes in DMRs associate with the GO term cartilage morphogenesis (Fig. 3C). This may be explained by the shared embryonic mesodermal origin of blood and the skeleton.

The DMP based hypergraph modelling approach employed here measures system-wide coordination of epigenomic features. In contrast to standard differential methylation analyses, this approach enables modelling KS1 and controls separately, revealing how the same elements of a system can be employed to different outcomes. We identified a subset of DMPs with higher order coordination of their methylation levels, indicating coordination in their function (Fig. 4). Interestingly, the hypergraph models of both KS1 and controls contain this same group of coordinated DMPs (Fig. 4C) suggesting similar functional relationships between differentially methylated CpGs in both groups. Despite this similarity, the hypergraph models show a difference in epigenomic network structure between groups, highlighted by a reduction in entropy within KS1 (Fig. 5). Lower entropy of omic networks is associated with ageing [75,76] and advancing cellular differentiation [52–54]. In addition, we observed that ontologies of genes with an identified DMR were markedly different to ontologies of either DMPs clustered in the hypergraph model, despite drawing from the same pool of CpGs, highlighting the unique insight gained from the hypergraph model.

Along with these DMPs, however, this approach highlighted a wider set of CpGs - hyperedges (shared correlations between any pair of DMPs) which were important in establishing the relationship between the coordinated DMPs. This analysis has highlighted possible disruption in microtubule formation which are essential for cell division, intracellular transport, and cell morphology and organization. This may affect the mechanical properties in the cell and in the nucleus as supported by Negri et al. [77]. These CpGs were not differentially methylated and as such, may contribute less directly to these phenotypes, however this observation would not have been possible through differential methylation analyses alone. Rather, the importance of these CpGs in the higher order organization of the epigenome may help to explain differences observed in individuals with genetic variants which are not accounted for by DMPs.

Gene ontology analysis may have limitations in DMP and DMR analysis as this emphasizes *cis-based* gene regulation. Hypergraph analysis factors in trans-regulatory gene regulation because as it uses the entire epigenome not just that present within genes or near genes. The overlap between hyperedges highlights chains of HOIs within the epigenome that may be considered as quantification of coordination and therefore a measurement of dysregulation rather than just the identification of directly associated disease related genes and pathways.

In conclusion, our study shows that the comparative analysis of peripheral blood DNA methylation reveals fundamental differences in the apparent functional activity of the epigenome. This study has identified novel candidate genes and pathways for KS1 disease pathology. Importantly, hypergraph approaches have not yet been used to assess mechanistic relationships and pathogenicity in single gene rare diseases. Our findings suggest that these approaches have the potential to reveal additional disease relevant mechanistic insights, which can be quantified, using epigenomes of rare disorders when compared with the traditional one-to-one differential analysis.

**Key Points**

- Generation of hypergraph models of epigenomic data, which capture complex relationships between many genes in rare conditions like KS1.
- Identification of biologically relevant pathways from DNA methylation data that escape the standard differentially methylated region-based analysis.
- Identification of coordination between DMPs, which revealed a wider variety of indirect associations of DMPs to be highly coordinated in KS1.
- This novel approach can be widely applied to different conditions to investigate mechanism in big data.

## Acknowledgments and funding sources

S.C., S.J.K., A.S., and S.B. acknowledge the support of Newlife Charity (grant number 16–17/10). This work was also supported by GOSH charity (grant number V4621), NIHR Manchester Biomedical Research Centre (NIHR203308 and the MRC Epigenomics of Rare Diseases Node (MR/Y008170/1) and ESPE fellowship (T.G.).

## Supplementary Material

Cuvertino_Garner_et_al_DNAm_KS_Supplementary_Figures_revision_bbae667
