# Supplementary material for: Higher order interaction analysis quantifies coordination in the epigenome revealing novel biological relationships in Kabuki syndrome
Source: Brief Bioinform. 2024 Dec 19;26(1):bbae667. doi: 10.1093/bib/bbae667 (PMC11658816; doi:10.1093/bib/bbae667)
Supplement: Cuvertino_Garner_et_al_DNAm_KS_Supplementary_Figures_revision_bbae667 [file cuvertino_garner_et_al_dnam_ks_supplementary_figures_revision_bbae667.pptx]

## Slide 1
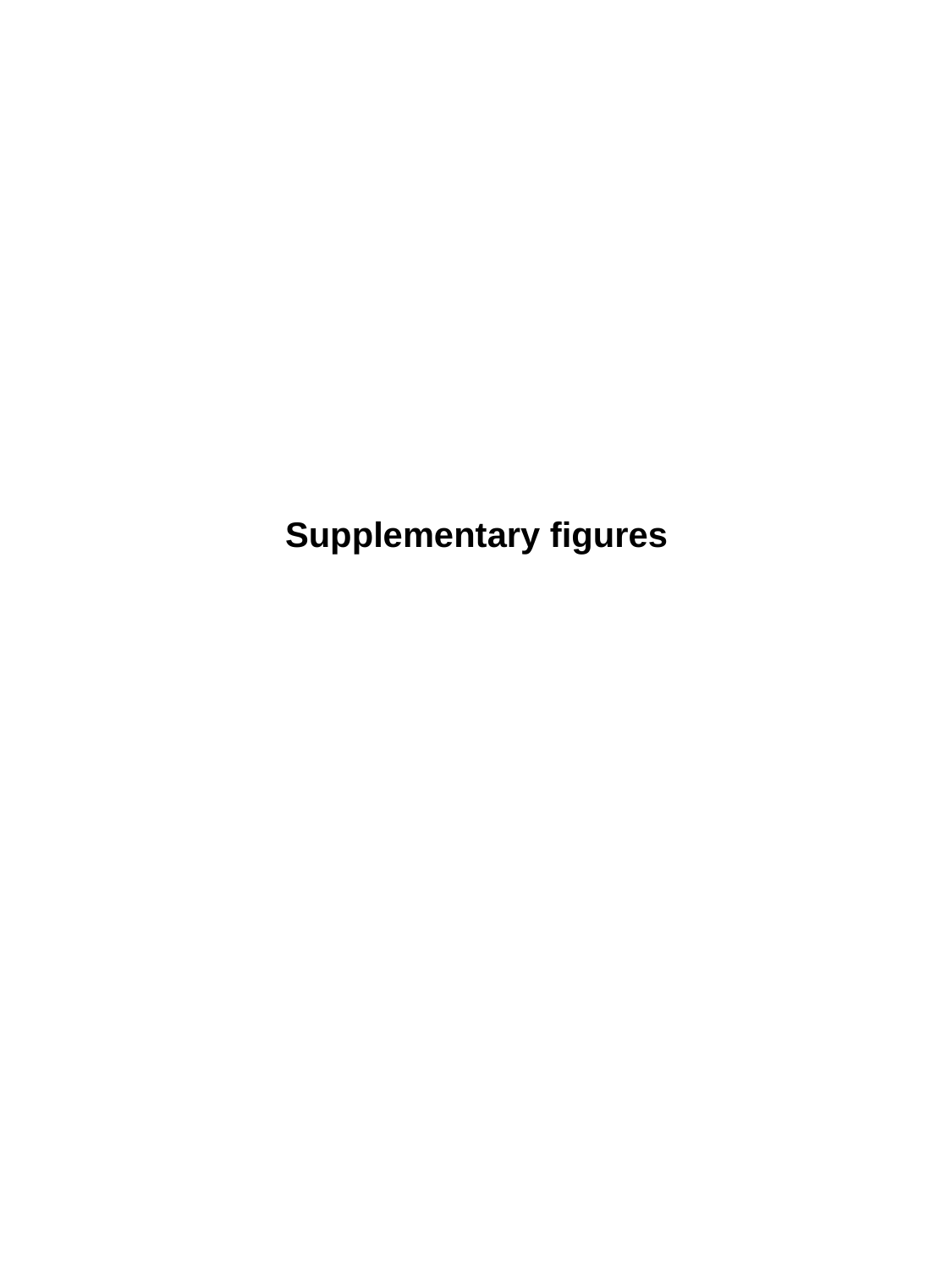

Supplementary figures

## Slide 2
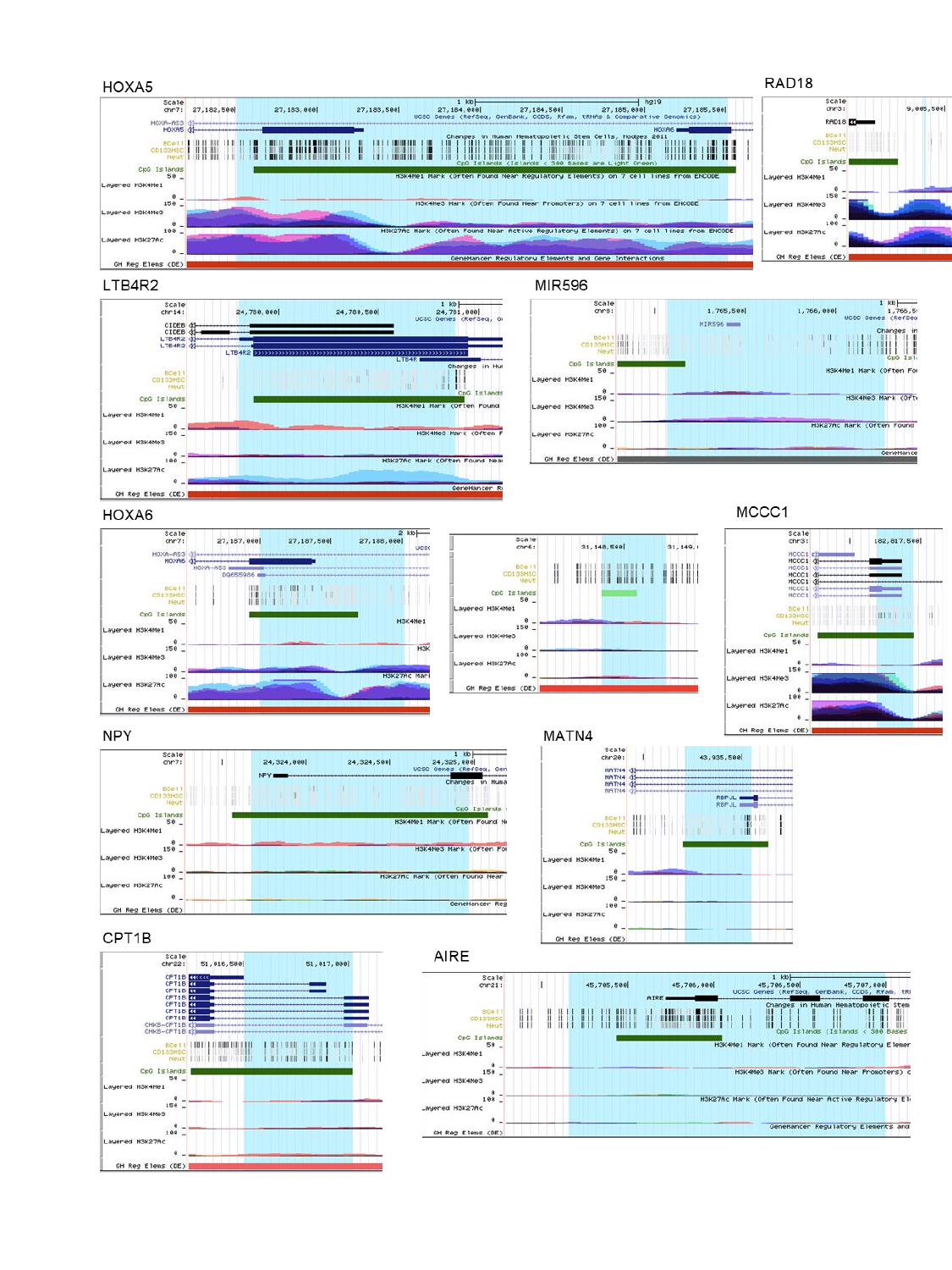

## Slide 3
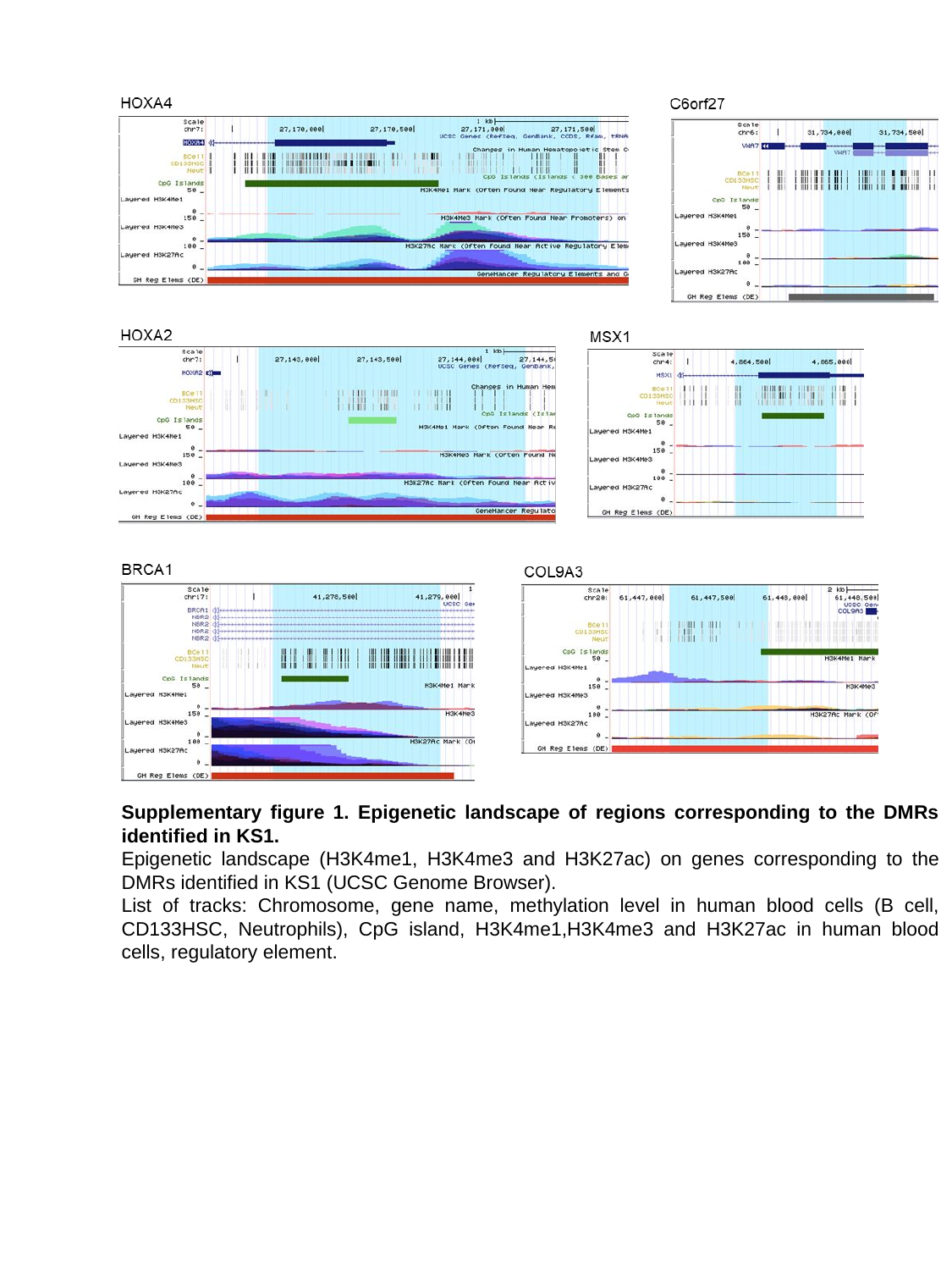

Supplementary figure 1. Epigenetic landscape of regions corresponding to the DMRs identified in KS1.
Epigenetic landscape (H3K4me1, H3K4me3 and H3K27ac) on genes corresponding to the DMRs identified in KS1 (UCSC Genome Browser).
List of tracks: Chromosome, gene name, methylation level in human blood cells (B cell, CD133HSC, Neutrophils), CpG island, H3K4me1,H3K4me3 and H3K27ac in human blood cells, regulatory element.

## Slide 4
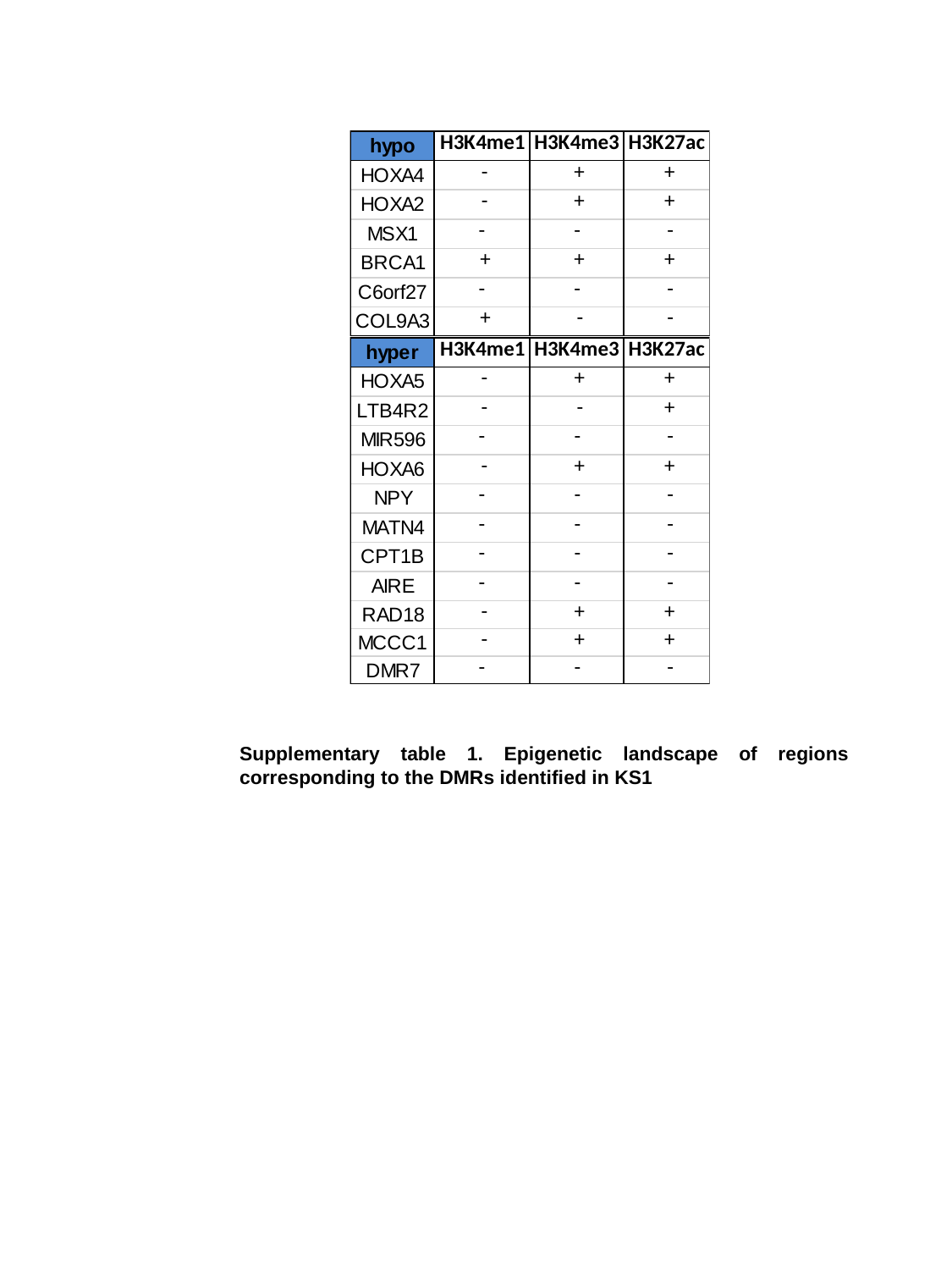

Supplementary table 1. Epigenetic landscape of regions corresponding to the DMRs identified in KS1

## Slide 5
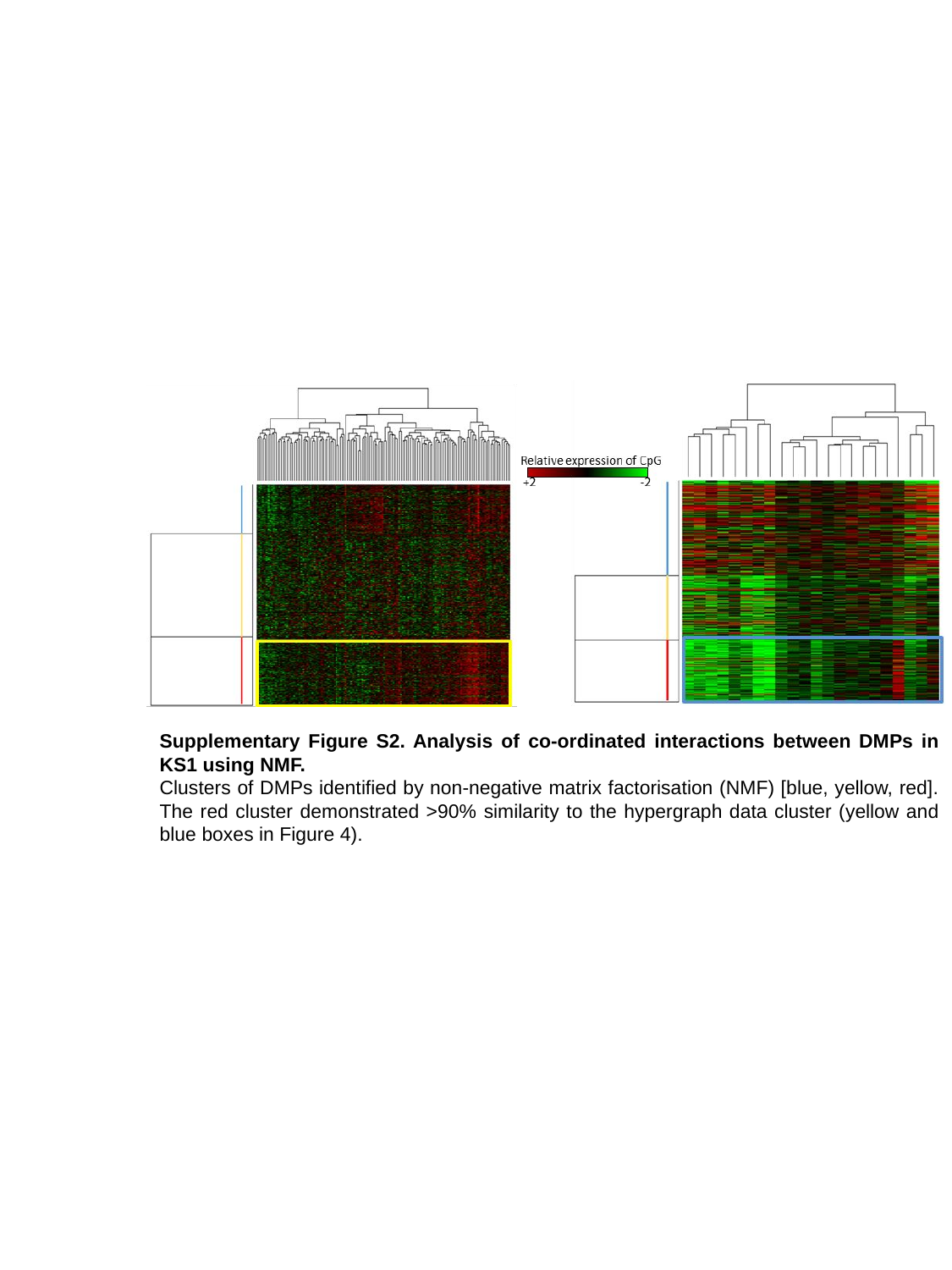

Supplementary Figure S2. Analysis of co-ordinated interactions between DMPs in KS1 using NMF.
Clusters of DMPs identified by non-negative matrix factorisation (NMF) [blue, yellow, red]. The red cluster demonstrated >90% similarity to the hypergraph data cluster (yellow and blue boxes in Figure 4).

## Slide 6
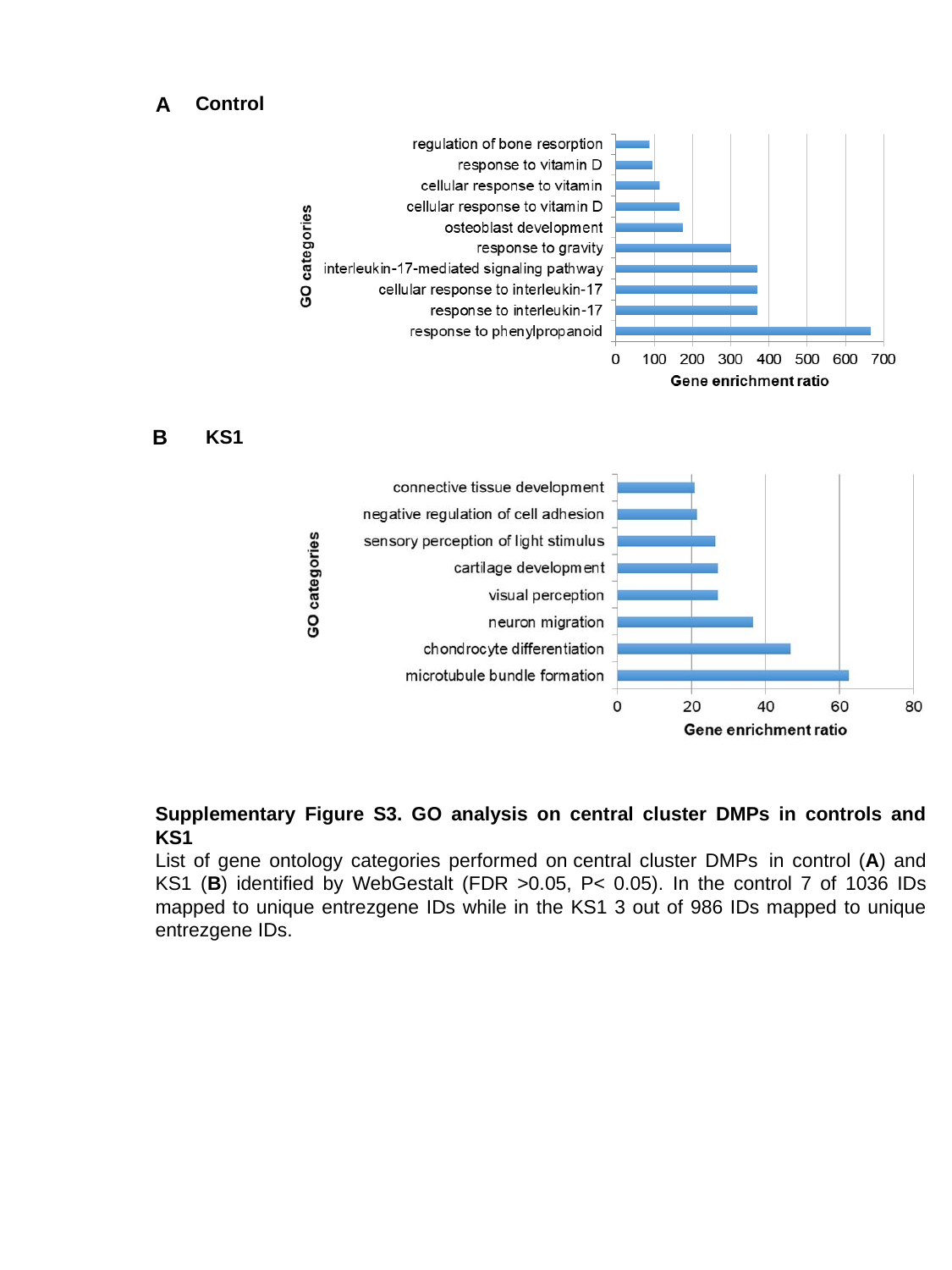

A
Control
B
KS1
Supplementary Figure S3. GO analysis on central cluster DMPs in controls and KS1
List of gene ontology categories performed on central cluster DMPs  in control (A) and KS1 (B) identified by WebGestalt (FDR >0.05, P< 0.05). In the control 7 of 1036 IDs mapped to unique entrezgene IDs while in the KS1 3 out of 986 IDs mapped to unique entrezgene IDs.

## Slide 7
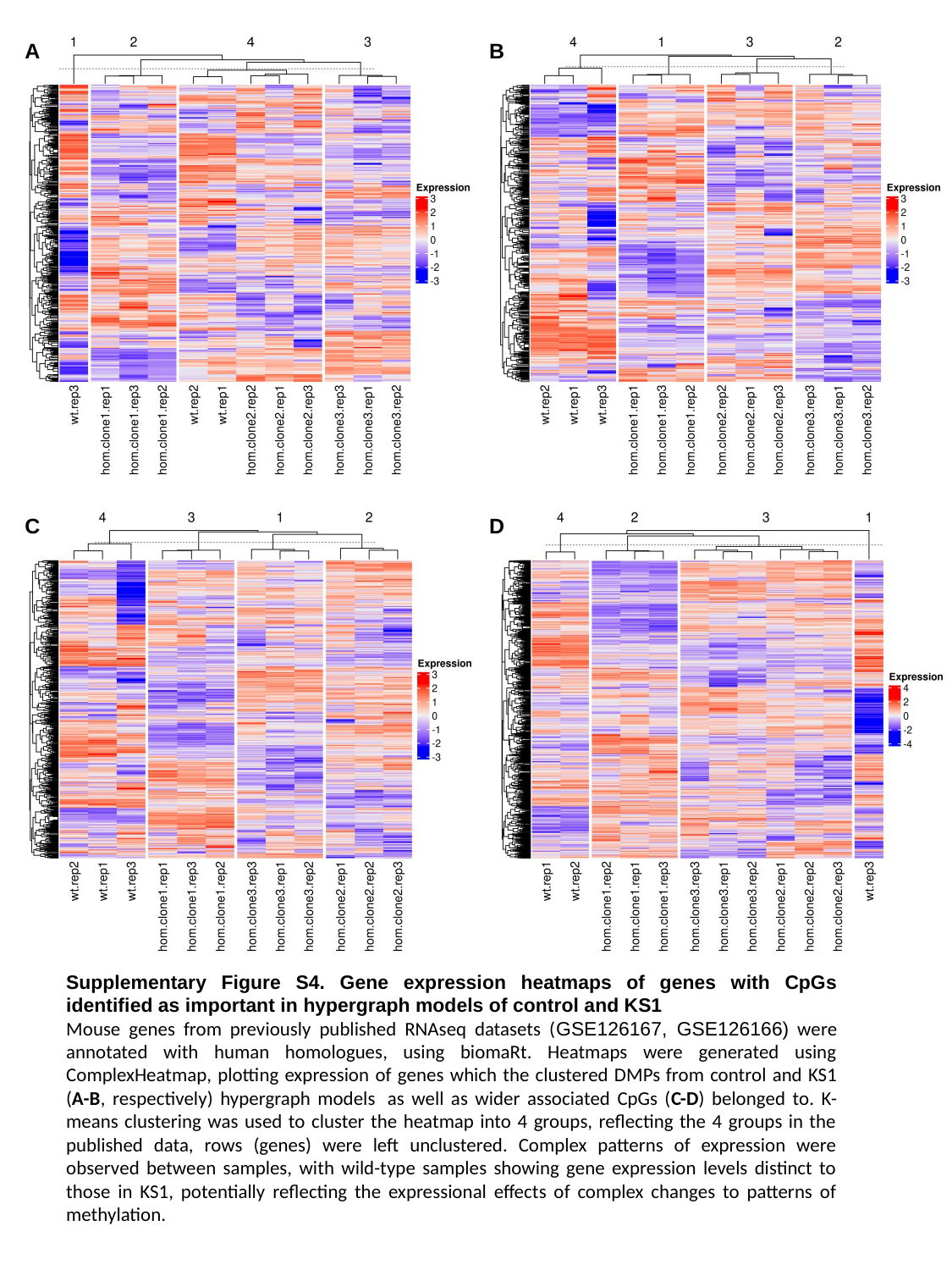

A
B
C
D
Supplementary Figure S4. Gene expression heatmaps of genes with CpGs identified as important in hypergraph models of control and KS1
Mouse genes from previously published RNAseq datasets (GSE126167, GSE126166) were annotated with human homologues, using biomaRt. Heatmaps were generated using ComplexHeatmap, plotting expression of genes which the clustered DMPs from control and KS1 (A-B, respectively) hypergraph models  as well as wider associated CpGs (C-D) belonged to. K-means clustering was used to cluster the heatmap into 4 groups, reflecting the 4 groups in the published data, rows (genes) were left unclustered. Complex patterns of expression were observed between samples, with wild-type samples showing gene expression levels distinct to those in KS1, potentially reflecting the expressional effects of complex changes to patterns of methylation.

## Slide 8
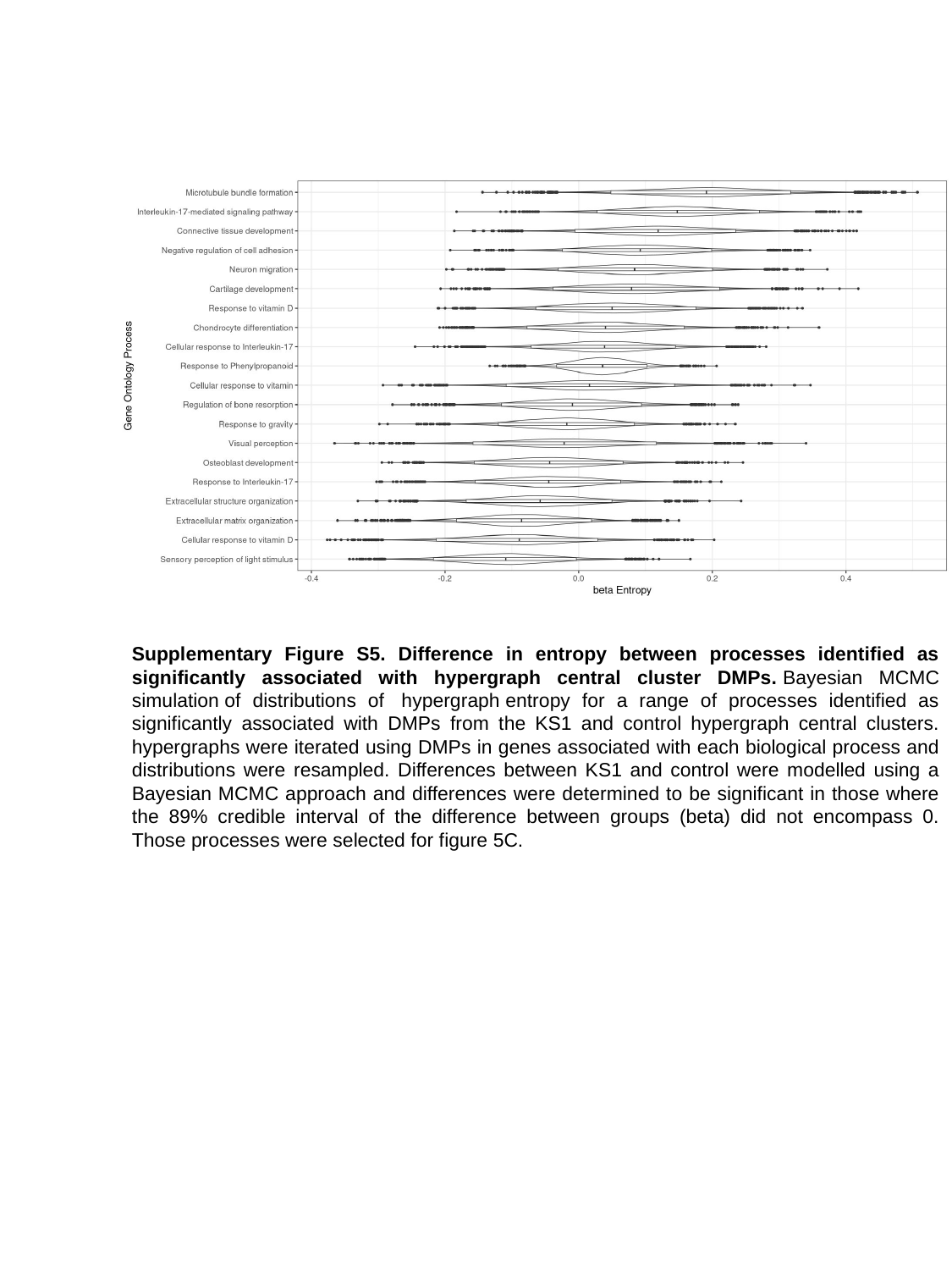

Supplementary Figure S5. Difference in entropy between processes identified as significantly associated with hypergraph central cluster DMPs. Bayesian MCMC simulation of distributions of  hypergraph entropy for a range of processes identified as significantly associated with DMPs from the KS1 and control hypergraph central clusters. hypergraphs were iterated using DMPs in genes associated with each biological process and distributions were resampled. Differences between KS1 and control were modelled using a Bayesian MCMC approach and differences were determined to be significant in those where the 89% credible interval of the difference between groups (beta) did not encompass 0. Those processes were selected for figure 5C.

## Slide 9
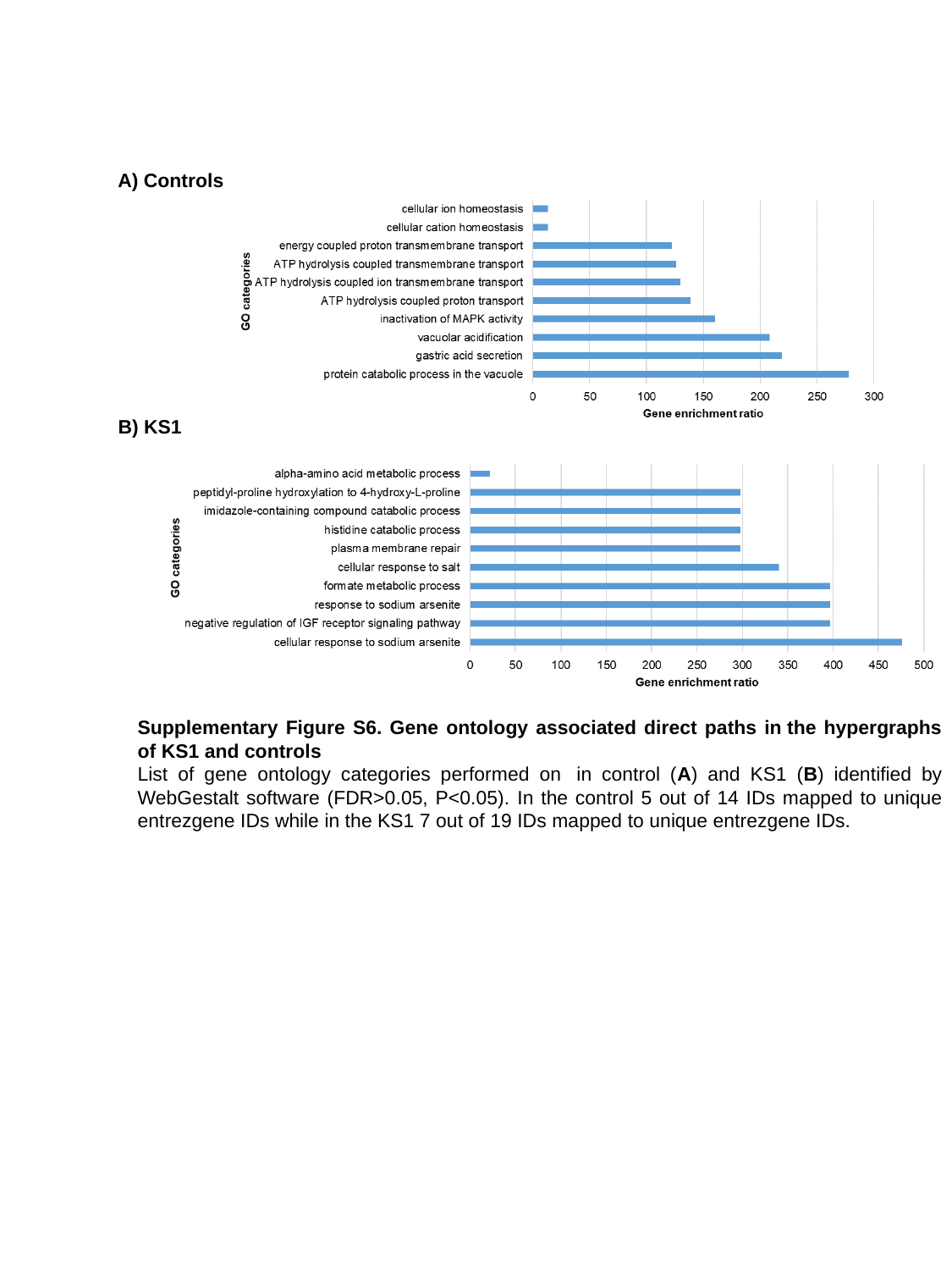

A) Controls
B) KS1
Supplementary Figure S6. Gene ontology associated direct paths in the hypergraphs of KS1 and controls
List of gene ontology categories performed on  in control (A) and KS1 (B) identified by WebGestalt software (FDR>0.05, P<0.05). In the control 5 out of 14 IDs mapped to unique entrezgene IDs while in the KS1 7 out of 19 IDs mapped to unique entrezgene IDs.

## Slide 10
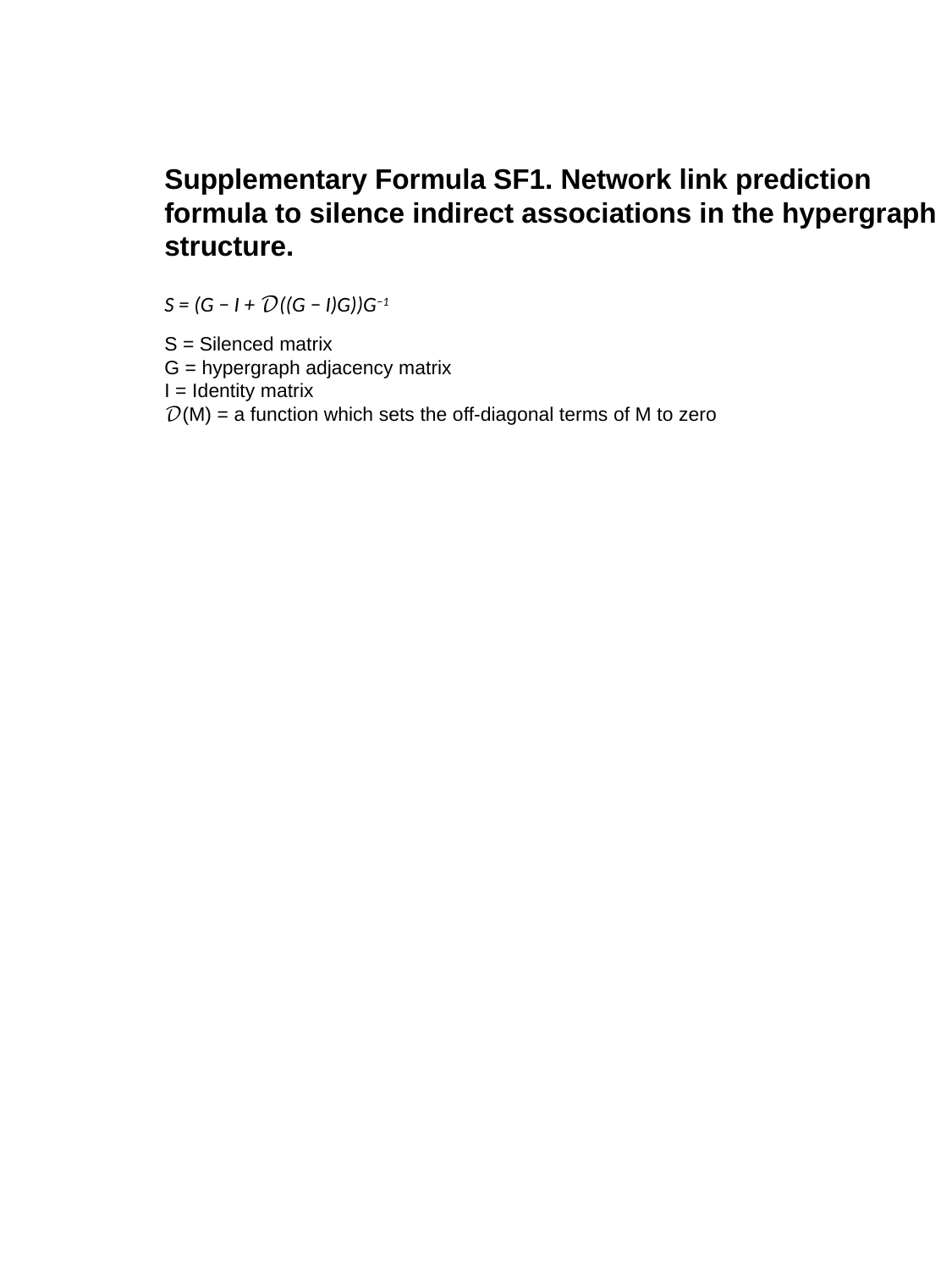

Supplementary Formula SF1. Network link prediction formula to silence indirect associations in the hypergraph structure.
S = Silenced matrix
G = hypergraph adjacency matrix
I = Identity matrix
𝒟(M) = a function which sets the off-diagonal terms of M to zero
S = (G − I + 𝒟((G − I)G))G−1
